# Supplementary material for: Kinesin-1 trans-synaptically regulates synaptic localization of SARM1 for asymmetric neuron diversification
Source: bioRxiv. 2026 Jan 6:2026.01.05.697830. Preprint. [Version 1] doi: 10.64898/2026.01.05.697830 (PMC12803128; doi:10.64898/2026.01.05.697830)
Supplement: Supplement 1 [file NIHPP2026.01.05.697830v1-supplement-1.pdf]

bioRxiv preprint doi: https://doi.org/10.64898/2026.01.05.697830; this version posted January 6, 2026. The copyright holder for this preprint (which was not certified by peer review) is the author/funder, who has granted bioRxiv a license to display the preprint in perpetuity. It is made available under aCC-BY 4.0 International license.

Supplemental Materials and Methods

The file includes supplemental strains and transgenes, plasmid construction, supplemental methods, and supplemental references.

bioRxiv preprint doi: https://doi.org/10.64898/2026.01.05.697830; this version posted January 6, 2026. The copyright holder for this preprint (which was not certified by peer review) is the author/funder, who has granted bioRxiv a license to display the preprint in perpetuity. It is made available under aCC-BY 4.0 International license.

Figure S1. (Related to Figure 4) *unc-116* mutations do not affect the expression levels of *odr-1p (-393-170)* in AWC. (A) Representative images of wild type and *unc-116(e2310)* animals expressing *odr-1p(-393-170)::GFP* in the AWC cell body taken at identical exposure times in the first larval stage. Scale bar, 5µm. Anterior to the left and ventral at the bottom. **(B)** Quantification of GFP fluorescence intensity in AWC cell bodies. *unc-116(e2310)* mutants displayed no significant difference in GFP intensity compared to wild type. Statistical analysis was performed using Student's *t*-test. Error bars, standard error of the mean. AU, arbitrary unit.

bioRxiv preprint doi: https://doi.org/10.64898/2026.01.05.697830; this version posted January 6, 2026. The copyright holder for this preprint (which was not certified by peer review) is the author/funder, who has granted bioRxiv a license to display the preprint in perpetuity. It is made available under aCC-BY 4.0 International license.

Figure S1

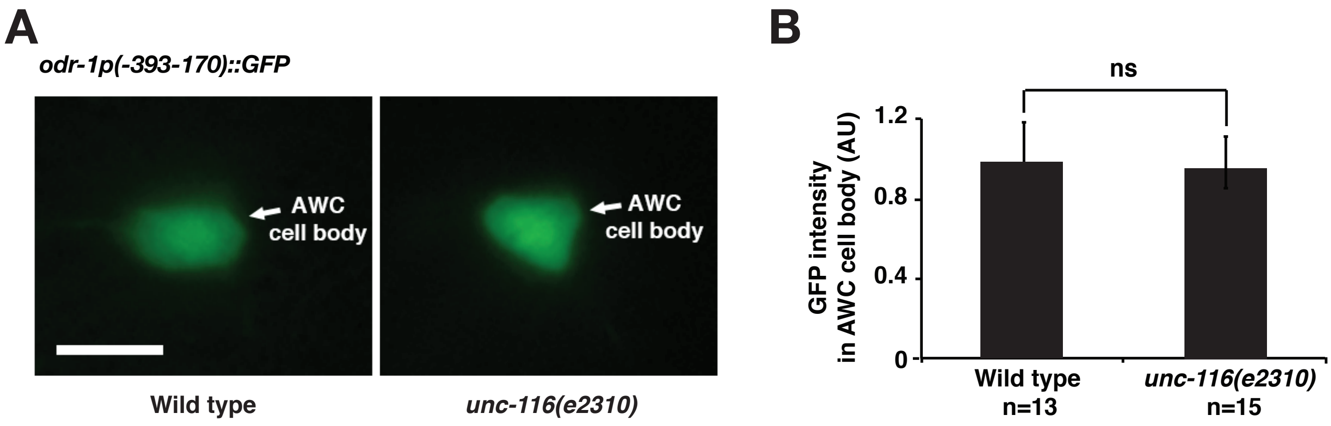

bioRxiv preprint doi: https://doi.org/10.64898/2026.01.05.697830; this version posted January 6, 2026. The copyright holder for this preprint (which was not certified by peer review) is the author/funder, who has granted bioRxiv a license to display the preprint in perpetuity. It is made available under aCC-BY 4.0 International license.

Supplemental Materials and Methods

Strains and transgenes

Animal protocols approved by the Office of Animal Care and Institutional Biosafety Committees at the University of Illinois Chicago were followed. Hermaphrodites of *C. elegans* were analyzed and imaged.

Mutants

unc-104 (e1265) II (HALL AND HEDGECOCK 1991)

tir-1 (ky388) III (CHUANG AND BARGMANN 2005)

unc-116 (e2310) III (PATEL *et al.* 1993)

unc-116 (rh24) III (PATEL *et al.* 1993)

unc-116 (rh24sb79) III (YANG *et al.* 2005)

Integrated transgenes

Integrated transgenes	Figures
kyIs140 [*str-2p::GFP; lin-15(+)*] I (TROEMEL *et al.* 1999)	1, 3
vyIs56 [*odr-1p::TagRFP*] III (ALQADAH *et al.* 2016)	2
duIs1 [*unc-116::GFP*] (MCNALLY *et al.* 2010)	2

Extrachromosomal arrays

Extrachromosomal arrays	Figures
vyEx2496, 2498 [*odr-3p::unc-116 (50 ng/ul); odr-1p::DsRed (15 ng/ul); ofm-1p::DsRed (30 ng/ul)*]	1, 3

bioRxiv preprint doi: https://doi.org/10.64898/2026.01.05.697830; this version posted January 6, 2026. The copyright holder for this preprint (which was not certified by peer review) is the author/funder, who has granted bioRxiv a license to display the preprint in perpetuity. It is made available under aCC-BY 4.0 International license.

vyEx2666 [*odr-3p::unc-116::GFP (1 ng/ul); odr-3p::tir-1::TagRFP (15ng/ul); ofm-1p::DsRed (30 ng/ul)*]	6
vyEx2714 [*odr-3p::unc-116::GFP (1 ng/ul); odr-3p::unc-104::TagRFP (15ng/ul); ofm-1p::DsRed (30 ng/ul)*]	2, 6
vyEx2473 [*odr-1p(-393-170)::tir-1::GFP (7.5 ng/ul); ofm-1p::DsRed (30 ng/ul)*]	4, 5
vyEx1609 [*odr-1p(-393-170)::GFP (50 ng/ul); ofm-1p::DsRed (30 ng/ul)*]	S1

Plasmid construction

odr-3p::unc-116 was generated by PCR amplifying a 3290 bp *unc-116* coding region from genomic DNA (Forward primer: GCACTAGCTAGCATGGAGCCGCGGACAG; Reverse primer: CATGCAGGTACCCTTGCTTGAAAAACTTTGATTAGTGAAAAATGGACG), which was then subcloned into a vector containing the *odr-3* promoter and *unc-54* 3' UTR.

odr-3p::unc-116::GFP was made by subcloning the *unc-116 gDNA* from *odr-3p::unc-116 gDNA* into a vector containing the *odr-3* promoter and *GFP* with *unc-54* 3' UTR.

odr-3p::tir-1::TagRFP was constructed by replacing GFP in *odr-3p::tir-1a cDNA::GFP* (CHUANG AND BARGMANN 2005) with TagRFP.

odr-3p::unc-104::TagRFP was made by subcloning 4752 bp of *unc-104* cDNA into the pCFJ356 vector (FRØKJÆR-JENSEN *et al.* 2012) containing the *odr-3* promoter and *TagRFP* with *unc-54* 3' UTR.

bioRxiv preprint doi: https://doi.org/10.64898/2026.01.05.697830; this version posted January 6, 2026. The copyright holder for this preprint (which was not certified by peer review) is the author/funder, who has granted bioRxiv a license to display the preprint in perpetuity. It is made available under aCC-BY 4.0 International license.

odr-1p(-393-170)::tir-1::GFP was generated by subcloning 2987 bp of *tir-1a* isoform cDNA into a vector containing the *odr-1(-393-170)* promoter and *GFP* with *unc-54* 3' UTR (ALQADAH *et al.* 2015).

Germline transformation

In brief, a DNA mix was injected into the syncytial gonad of adult hermaphrodites (P_0) as previously described (MELLO AND FIRE 1995). F_1 animals expressing the co-injected fluorescent transgenes were identified and cloned (1 animal per plate), and the F_2 progenies were screened and selected for transgenic lines.

Genetic mosaic analysis

Animals containing unstable extrachromosomal transgene arrays were passed for at least six generations before performing mosaic analysis, as previously described (SAGASTI *et al.* 2001; VANHOVEN *et al.* 2006). The co-injection marker *odr-1p::DsRed* (expressed in both AWC neurons) was used to identify the mosaic animals that lose the extrachromosomal transgene in one of the two AWC cells.

Live imaging of transgenic animals expressing fluorescent proteins

Animals were mounted onto 2% agarose pads and anesthetized with 5 mM sodium azide (Sigma) or 7.5 mM levamisole (Sigma). Z-stack images were obtained using a Zeiss Axio Imager M2 microscope, equipped with a motorized focus drive, a Zeiss objective EC Plan-Neofluar 40x/1.30 Oil DIC M27, a Piston GFP bandpass filter set (41025, Chroma Technology), a TRITC filter set (41002c, Chroma Technology), a Hamamatsu digital camera C11440, and a Zeiss Apotome system. Images were acquired using Zeiss imaging software, ZEN (2012 Blue Edition SP2).

Quantification of fluorescence intensity

bioRxiv preprint doi: https://doi.org/10.64898/2026.01.05.697830; this version posted January 6, 2026. The copyright holder for this preprint (which was not certified by peer review) is the author/funder, who has granted bioRxiv a license to display the preprint in perpetuity. It is made available under aCC-BY 4.0 International license.

Animals for each set of experiments were imaged using the same exposure time. Fluorescence intensity was measured using the Zeiss imaging software ZEN. In Figure S1, a single focal plane with the brightest GFP fluorescence in the AWC cell body was selected from Z-stack images to compare fluorescence intensity. The quantification analysis was performed by the same individual.

Time-lapse imaging of protein trafficking

Time-lapse imaging was performed as previously described (CHANG *et al.* 2011; SIETE *et al.* 2024). Worms in the second larval stage were anesthetized with 7.5 mM tetramisole and mounted onto 2% agarose pads on microscope slides for imaging. We previously showed that 7.5 mM tetramisole, compared to 0.5 mM, 1 mM, and 2 mM tetramisole, did not significantly affect the axonal transport of TIR-1::GFP along the AWC axons (SIETE *et al.* 2024). Time-lapse images were acquired for 30 seconds with an exposure time of 300 milliseconds and a speed of 3 frames per second using a Zeiss Axio Imager M2 microscope, equipped with a Zeiss objective EC Plan-Neofluar 63x/1.40 Oil DIC M27, a Piston GFP bandpass filter set (41025, Chroma Technology), a Hamamatsu digital camera C11440, and the Zeiss imaging software ZEN (2012 blue edition SP2). Acquired images were analyzed to generate kymographs using the Fiji software (SCHINDELIN *et al.* 2012). The percentage and velocity of moving events were analyzed using ImageJ software.

Supplemental references

Alqadah, A., Y. W. Hsieh, J. A. Schumacher, X. Wang, S. A. Merrill *et al.*, 2016 SLO BK Potassium Channels Couple Gap Junctions to Inhibition of Calcium Signaling in Olfactory Neuron Diversification. PLoS Genet 12**:** e1005654.

Alqadah, A., Y. W. Hsieh, B. Vidal, C. Chang, O. Hobert *et al.*, 2015 Postmitotic diversification of olfactory neuron types is mediated by differential activities of the HMG-box transcription factor SOX-2. The EMBO Journal 34**:** 2574-2589.

bioRxiv preprint doi: https://doi.org/10.64898/2026.01.05.697830; this version posted January 6, 2026. The copyright holder for this preprint (which was not certified by peer review) is the author/funder, who has granted bioRxiv a license to display the preprint in perpetuity. It is made available under aCC-BY 4.0 International license.

Chang, C., Y.-W. Hsieh, B. J. Lesch, C. I. Bargmann and C.-F. Chuang, 2011 Microtubule-based localization of a synaptic calcium-signaling complex is required for left-right neuronal asymmetry in C. elegans. Development 138**:** 3509-3518.

Chuang, C. F., and C. I. Bargmann, 2005 A Toll-interleukin 1 repeat protein at the synapse specifies asymmetric odorant receptor expression via ASK1 MAPKKK signaling. Genes Dev 19**:** 270-281.

Frøkjær-Jensen, C., M. W. Davis, M. Ailion and E. M. Jorgensen, 2012 Improved Mos1-mediated transgenesis in C. elegans. Nature Methods 9**:** 117-118.

Hall, D. H., and E. M. Hedgecock, 1991 Kinesin-related gene unc-104 is required for axonal transport of synaptic vesicles in C. elegans. Cell 65**:** 837-847.

McNally, K. L., J. L. Martin, M. Ellefson and F. J. McNally, 2010 Kinesin-dependent transport results in polarized migration of the nucleus in oocytes and inward movement of yolk granules in meiotic embryos. Developmental Biology 339**:** 126-140.

Mello, C., and A. Fire, 1995 DNA transformation. Methods in Cell Biology 48**:** 451-482.

Patel, N., D. Thierry-Mieg and J. R. Mancillas, 1993 Cloning by insertional mutagenesis of a cDNA encoding Caenorhabditis elegans kinesin heavy chain. Proceedings of the National Academy of Sciences 90**:** 9181-9185.

Sagasti, A., N. Hisamoto, J. Hyodo, M. Tanaka-Hino, K. Matsumoto *et al.*, 2001 The CaMKII UNC-43 activates the MAPKKK NSY-1 to execute a lateral signaling decision required for asymmetric olfactory neuron fates. Cell 105**:** 221-232.

Schindelin, J., I. Arganda-Carreras, E. Frise, V. Kaynig, M. Longair *et al.*, 2012 Fiji: an open-source platform for biological-image analysis. Nature Methods 9**:** 676-682.

Siete, C., R. Xiong, A. Khalid, Y.-W. Hsieh and C.-F. Chuang, 2024 Immobilization of C. elegans with different concentrations of an anesthetic for time-lapse imaging of dynamic protein trafficking in neurons. MicroPubl Biol.

bioRxiv preprint doi: https://doi.org/10.64898/2026.01.05.697830; this version posted January 6, 2026. The copyright holder for this preprint (which was not certified by peer review) is the author/funder, who has granted bioRxiv a license to display the preprint in perpetuity. It is made available under aCC-BY 4.0 International license.

Troemel, E. R., A. Sagasti and C. I. Bargmann, 1999 Lateral Signaling Mediated by Axon Contact and Calcium Entry Regulates Asymmetric Odorant Receptor Expression in C. elegans. Cell 99**:** 387-398.

Vanhoven, M. K., S. L. Bauer Huang, S. D. Albin and C. I. Bargmann, 2006 The claudin superfamily protein nsy-4 biases lateral signaling to generate left-right asymmetry in C. elegans olfactory neurons. Neuron 51**:** 291-302.

Yang, H.-y., P. E. Mains and F. J. McNally, 2005 Kinesin-1 mediates translocation of the meiotic spindle to the oocyte cortex through KCA-1, a novel cargo adapter. The Journal of Cell Biology 169**:** 447-457.
